# Adverse Health Effects of Thirdhand Smoke: From Cell to Animal Models

**DOI:** 10.3390/ijms18050932

**Published:** 2017-04-28

**Authors:** Bo Hang, Pin Wang, Yue Zhao, Altaf Sarker, Ahmed Chenna, Yankai Xia, Antoine M. Snijders, Jian-Hua Mao

**Affiliations:** 1 Biological Systems and Engineering Division, Lawrence Berkeley National Laboratory, Berkeley, CA 94720, USA; pinwang@lbl.gov (P.W.); yuezhao@lbl.gov (Y.Z.); ahsarker@lbl.gov (A.S.); AMSnijders@lbl.gov (A.M.S.); jhmao@lbl.gov (J.-H.M.); 2 Department of Gastroenterology, Drum Tower Clinical Medical School of Nanjing Medical University, Nanjing 210008, China; 3 LabCorp Specialty Testing Group, Monogram Biosciences Inc., South San Francisco, CA 94080, USA; a_chenna@yahoo.com; 4 State Key Laboratory of Reproductive Medicine, Institute of Toxicology, Nanjing Medical University, Nanjing 211166, China; yankaixia@njmu.edu.cn

**Keywords:** thirdhand smoke, secondhand smoke, DNA damage, DNA strand breaks, DNA adducts, genotoxicity, early exposure, animal studies, health impact, tobacco control

## Abstract

The newly identified smoke hazard, thirdhand smoke (THS), has gained public attention in recent years but its health impact and biological effects are largely unknown. THS may be defined by “the four Rs”: tobacco chemicals that remain, react, re-emit, and/or are resuspended long after active smoking has ceased. This review summarizes recent research progress in the effects of THS on genotoxicity, metabolism and early life development using cellular and animal models. We first reported that THS generated in laboratory systems caused significant DNA damage in human cell lines. Our finding that THS significantly induces oxidative base lesions has been confirmed in skin wounds of mice models exposed to THS. THS also induced metabolomic changes in human reproductive cell lines. Furthermore, we demonstrated that early exposure to THS not only negatively impacts body weight in both male and female mice, but also induces persistent changes to immunological parameters in peripheral blood in these mice. These results indicate that THS is genotoxic at realistic experimental doses and that there may be a window of susceptibility for some forms of cellular damage induced by THS.

## 1. Thirdhand Smoke: The Concept and Features

Mainstream smoke (MSS), or firsthand smoke, is created by tobacco combustion at 600–900 °C, when smokers inhale the smoke from a burning cigarette, whereas sidestream smoke (SSS) emanates from the smoldering end of a lit cigarette at ~600 °C between puffs. Secondhand smoke (SHS), or environmental smoke, is a mixture of SSS (~85%) and exhaled MSS (~15%) [1]. There have been considerable amounts of research on the toxicity and health impact of active smoking and passive smoking. Both have been causally linked to a wide range of diseases and other adverse consequences, as well documented in the Surgeon General Reports of 1964 on active smoking [2] and of 1986 on passive smoking (in the form of SHS) [3]. In recent years, a “hidden” risk from tobacco smoke has been revealed, that is, SHS that lingers in the indoor environment over many hours and may become more hazardous with time [4], suggesting that people are at risk from exposure to cigarette smoke residues in ways that have not been recognized before. This so-called thirdhand smoke (THS) [5] is defined as the contamination of surfaces in contact with compounds emitted in SHS, the novel products generated by chemical transformations of the components, and the off-gassing of volatile components into the air. The phrase “the four Rs” provides a working definition of THS: tobacco chemicals that remain, react, re-emit, and/or are resuspended long after active smoking has ceased [6,7]. It is commonly known that exposure to SHS is associated with freshly emitted smoke and the primary pathway for exposure is inhalation within a relatively short time (normally minutes to a few hours). In contrast, exposure pathways for THS include not only inhalation but also ingestion and dermal contact. However, the exposure to THS is much longer than SHS and could last weeks and months [6,7]. THS contamination can be pervasive, as shown in studies performed in the U.S. [7], and appears to be much worse in places with heavy smoking, as clearly shown in our previous studies carried out in the city of Nanjing, China [8].

THS is a form of aged SHS. Interestingly, the “aging process” of SHS has become a focus of recent studies with regard to its physical chemistry and toxicological property. Studies led by the Lawrence Berkeley National Laboratory [9,10] have demonstrated that compounds in SHS sorbed onto indoor surfaces can react with common indoor pollutants to produce new and even more toxic species [9,10]. One example of such chemical transformation in SHS is the reaction of surface-bound nicotine with indoor ozone to generate toxic compounds such as formaldehyde and *N*-methylformamide [9]. Nicotine is a main constituent in THS with a high emission rate, high concentrations and persistence on indoor surfaces [7]. Another case is the reaction of nicotine with indoor pollutant nitrous acid (HONO) to generate substantial levels of tobacco-specific nitrosamines (TSNAs), including NNK (4-(methylnitrosamino)-1-(3-pyridyl)-1-butanone), NNN (*N*′-nitrosonornicotine) and NNA (1-(*N*-methyl-*N*-nitrosamino)-1-(3-pyridinyl)-4-butanal) [10]. Both NNK and NNN are known potent human carcinogens [11]. NNA is mainly found in THS, and rarely detected in MSS or SSS. Not much is reported about the toxicity of NNA [12] which has been the research interest of our studies in recent years (see below). Substantial levels of TSNAs were measured on surfaces inside households (hundreds of nanograms per square meter), smoker’s skins, clothes and vehicles, including the major product NNA, and they build up further with frequent smoking. It should be mentioned that recent studies revealed that THS contains many classes of toxic compounds, including both semi-volatile organic compounds (SVOCs) and volatile organic compounds (VOCs) [10,13]. Some of these compounds are known tobacco toxins and carcinogens [12].

**Figure 1:**
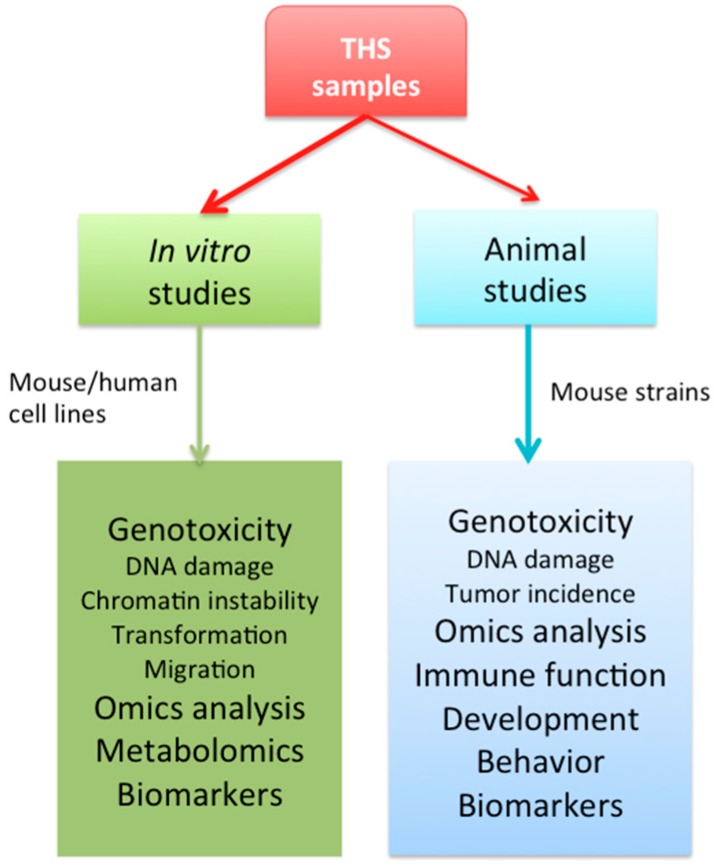
Schematic representation of the main approaches and models proposed for current and future studies on thirdhand smoke (THS)-induced biological and health effects.

This overview summarizes the scientific findings on THS-induced biological effects and health impact made in the authors’ laboratories using in vitro and animal models, and also including those from collaborations with other researchers (Figure 1).

## 2. Exposure to Thirdhand Smoke (THS) Is Genotoxic

Genotoxicity is one of the critical mechanisms responsible for many types of cancer caused by active smoking and SHS exposure. As mentioned above, similar to SHS, THS contains many toxic compounds, including known mutagens and carcinogens as defined by the International Agency for Research on Cancer (IARC) and National Toxicology Program (NTP). However, when the concept of THS was first revealed, its genotoxic potential, a critical aspect in risk assessment, was completely unknown. This prompted us to investigate the DNA damaging capacity of THS and its constituent(s), as described below.

### 2.1. THS Induces DNA Strand Breaks in Human Cells

The generation of standardized THS materials is a critical aspect in toxicological studies. THS-laden materials were produced in well-controlled laboratory systems located either at Lawrence Berkeley National Laboratory (LBNL) or University of California, San Francisco (UCSF), controlling smoke concentration, flow rate, time, substrate, and storage conditions. Sample extracts were analyzed with liquid chromatography–tandem mass spectrometry (LC–MS/MS) for common THS components including, for example, nicotine, cotinine and TSNA [14].

The genotoxic potential of THS and its known constituents was for the first time assessed in human cell lines using the alkaline Comet assay in 2013 [14]. Double-strand break (DSB) is the most lethal type of DNA damage, as both strands of the DNA duplex are compromised. Significant increases in DNA strand breaks were observed in human liver cancer cells (HepG2) exposed to THS. Both acutely and chronically perfused THS materials induced DNA strand breaks, although chronically perfused materials induced more DNA damage than acutely perfused materials. The concentrations of THS components in the THS perfused materials were realistic and close to real world pollution levels. Most recently, utilizing the DNA damage markers γ-H2AX and p53BP1 co-localization approach, we confirmed the formation of DSBs in human lung epithelial cells (BEAS-2B) following exposure to THS [15]. The results are also comparable to the number of DSBs formed after exposure to SHS [15]. Failure in appropriate DSB repair could lead to an increase in cancer risk by inducing genomic instability.

### 2.2. THS Induces Oxidative DNA Damage in Human Cells and Animal Models

We have recently reported significantly higher levels of oxidative DNA damage in both *HPRT* (coding hypoxanthine-guanine phosphoribosyltransferase) and *POLB* (coding DNA polymerase β) genes in BEAS-2B cells exposed to THS. For this study we used the long amplicon-qPCR (LA-qPCR) assay [14], which is highly sensitive to oxidative DNA damage when coupled with Fpg/Nei glycosylases that are specific for incision of oxidized bases. *HPRT* and *POLB* genes were used as representative genes, and also Van Houten’s group had already standardized the conditions for the LA-qPCR assay with these two genes [16]. Our results suggest that THS exposure may cause oxidative damage in DNA that could be an important contributing factor in THS-mediated cellular toxicity.

To further confirm the effect of THS on the accumulation of oxidative DNA lesions, we measured the oxidative stress-induced DNA damage in mouse skin wounds exposed to THS using the same LA-qPCR assay. We found increased levels of oxidative DNA damage in mouse *Polβ* and *β-globin* genes [17]. This finding was in agreement with high levels of 8-oxo-dGuo identified in the same tissues. Oxidative DNA damage can lead to disease causing mutations, such as in cancer.

### 2.3. 1-(N-Methyl-N-Nitrosamino)-1-(3-Pyridinyl)-4-Butanal (NNA) Exposure Causes the Formation of DNA Adducts

It is well accepted that formation of DNA adducts, especially bulky adducts, plays a central role in smoking-induced mutagenesis and carcinogenesis [12]. If DNA adducts persist unrepaired, they can lead to mutation. The role of NNK as a potent lung carcinogen has been well studied, and NNK is known to form adducts with DNA in vivo, including bulky pyridyloxobutyl (POB) adducts [18]. The latter are at higher levels in lung tissue of smokers compared to nonsmokers, as judged by the levels of 4-hydroxy-1-(3-pyridyl)-1butanone (HPB) releasing-DNA adducts [19]. As for NNA, its ability to cause DNA damage is poorly understood. To gain insight, we first used the Comet assay to show that NNA was able to cause dose-dependent DNA strand breaks in HepG2 cultures. These cells were exposed to NNA at non-cytotoxic concentrations ranging from 0.01 to 100 µM for 24 h [14].

With the use of liquid chromatography-electrospray ionization-tandem mass spectrometry (LC-ESI–MS/MS) and two-dimensional nuclear magnetic resonance spectroscopy (2D NMR), we identified for the first time five different adducts formed from the in vitro reaction of NNA with 2’-deoxyguanosine (dGuo) [15]. In addition to *N*^1^-methyl-dGuo*O*^6^-methyl-dGuo and 8-oxo-dGuo, we also identified two modifications that are novel in structure: (1) 1,*N*^2^-NNA-dGuo, from the condensation of NNA and dGuo with the elimination of H_2_O and two H molecules (addition of neutral C_10_H_9_N_3_O to dGuo). Its chemical structure is proposed based on ESI–MS/MS and 2D NMR [7]. Given that NNA is highly selective for THS, this covalent bulky adduct would be a promising biomarker for exposure; (2) 5′,3′-dimethyl-dGuo which is a novel sugar damage that may lead to the breakage of the DNA backbone if it is formed in THS-exposed cells. 

## 3. THS Exposure Causes Metabolomic Changes in Reproductive Cells

Exposure to chronic THS samples in two rodent male reproductive cell lines, GC-2 and TM-4, caused significant alterations in the metabolome [20]. We demonstrated that at low THS concentrations that yielded normal cell viability, cell cycle, apoptosis, and reactive oxygen species (ROS) production, glutathione metabolism in GC-2 cells and nucleic acid and ammonia metabolism in TM-4 cells were altered significantly. In addition, RT-PCR analyses of mRNAs for enzyme genes showed changes in the expression levels of genes that encode enzymes involved in glutathione, nucleic acid, and ammonia metabolism. A metabolomic approach could help identify biomarkers for exposure and risk assessment in THS-related research.

## 4. Early Life THS Exposure Affects Body Mass and the Development of Immunity in Mice

The concept of THS as a distinct entity that poses health risks for small children has developed only recently. Since infants typically spend more time indoors and have age-specific behaviors, i.e., crawling and ingesting non-food items, they are often in close contact with surfaces and dust. Moreover, children are more sensitive than adults to pollutants for several reasons, including increased respiration rate/body size; immaturity of immunologic systems; and low metabolic capacity. Thus, even low doses of THS constituents may represent a long-term health hazard to them. It was reported by Matt et al. [21] that the homes of parents who only smoked outdoors still had higher levels of nicotine than the homes of nonsmokers and, more importantly, the children of smoking parents who never smoked in the home had higher urinary cotinine levels than the children of nonsmokers. Even in places where smoking bans are strictly enforced, such as neonatal intensive care units in hospitals, THS can be found by measuring (4-(methylnitrosamino)-1-(3-pyridyl)-1-butanol) (NNAL)/cotinine concentrations in infants’ urine [22]. By analyzing nicotine and nitrosamines/TSNAs in house samples, Ramirez et al. found that the calculated cancer risk for children (1 to 6 years old) increased [23]. In addition, low level prenatal exposure of tobacco smoke correlates with cognitive deficits in infants, suggesting that some compounds in tobacco smoke may be neurotoxic [24].

Although these previous studies suggest that THS is a potential health threat to infants and young children who are in smokers’ homes, virtually nothing is known about the specific health effects of THS exposure at these stages. We have recently studied the effects of THS exposure on body mass and blood cell populations from birth until weaning (postnatal day 21) in C57BL/6J mice [25]. One finding of this study was a significant reduction in weight gain in both THS-treated male and female mice as compared to the non-exposed mice. When the mice were removed to a non-exposed setting and followed over time, the body weight of THS-exposed mice caught up to control mice at 5, 8, 12 and 17 weeks.

We also found that at 17 weeks of age, in the adult male and female mice exposed to THS during the first 3 weeks of life, the eosinophil number in blood circulation was significantly higher in both genders, together with increased basophils in male mice and increased neutrophils in female mice. The number of specific types of immune cells was significantly changed. Fluorescence-activated cell sorting (FACS) analysis showed a significantly increased percentage of B-cells and T-suppressor cells, with a decreased percentage of myeloid cells in adult mice [18]. These results indicate that there may be a window of susceptibility for some forms of cellular damage induced by THS exposure. The damage that occurs during the very early stages of life may persist into adulthood.

It should be noted that a number of other laboratories have extensively studied the toxicity of THS or its components using various models and methods, together with analysis of chemical changes in THS samples. These include studies on important cellular processes in various types of cells, including stem cells [26,27,28,29,30,31,32,33], animal-based research [34,35,36], and human health impact [23]. Majority of these studies have been discussed in the recent review by Jacob et al. [7]. Therefore, detailed descriptions of these areas were not covered in this overview.

## 5. Summary and Perspective

THS is a new hidden health hazard, with infants and children being most at risk of higher exposure. We have shown that: (1) In cultured human cells and animal skins, THS exposure induces DNA damage, including DNA strand breaks and oxidative DNA damage, and metabolomic changes; (2) NNA causes the formation of DNA adducts, including 1, *N*^2^-NNA-dGuo; (3) In newborn mice, early-exposure to THS caused significant body weight change and long-lasting immunological changes. Systematic in vitro and in vivo studies together with recently developed biotechnologies (such as next generation sequencing) will need to be performed to validate and extend our current knowledge of THS-related health effects using additional cell lines and animal models.

**Figure 2:**
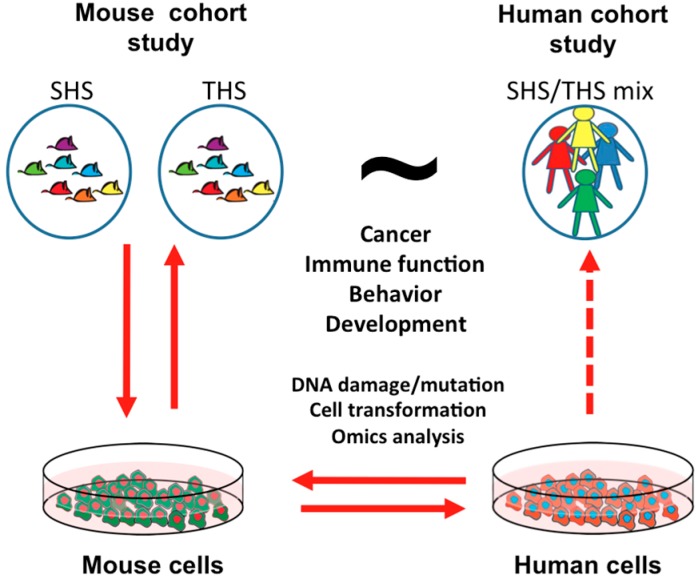
Parallelogram strategy that will employ integrative mouse and human systems biology to investigate long-term health effects of exposure to THS. Solid arrows indicate that the data support each other. The dashed arrow on the right indicates that the results from the cell system can infer the human population-based study.

We propose that an integrative systems biology approach will provide a comprehensive evaluation of the biological and health effects of exposure to THS. Since direct human studies linking THS to human risks and diseases are virtually impossible, model systems, such as mouse models, will serve as valuable tools. Since infants and toddlers living in a smoking household are normally exposed to mixed SHS and THS, mouse studies can separately study the effects of SHS and THS exposure in a well-controlled environment, as shown in Figure 2. The animal studies should be coordinated with in vitro experiments, e.g., comparing effective concentrations in vitro to serum or tissue levels in animals and comparing the effects between mouse and human cells. This “parallelogram strategy” capitalizes on the strengths of complementary in vivo and in vitro mouse and human systems, especially the use of well-defined human cohort studies (see Figure 2).

The identification and measurement of biomarkers for THS exposure is also a key research area. We have been focusing on NNA and its metabolites/DNA adducts to explore whether they can be likely candidates for biomarkers for THS exposure. For example, the detection of NNA-derived DNA covalent adducts in blood cells may serve as an early indicator of THS exposure and harm.

Taken together, the knowledge and results obtained from these studies are expected to generate novel information to understand the effects of THS and translate them into human and clinical implications that should lead to new strategies for prevention of THS-related diseases.

## Abbreviations

THS	Thirdhand smoke
SHS	Secondhand smoke
SSS	Mainstream smoke
DSB	Double-strand break
dGuo	2′-Deoxyguanosine
dCyt	2′-Deaxycytidine
IARC	International Agency for Research on Cancer
NTP	National Toxicology Program
HONO	Nitrous acid
TSNA	Tobacco-specific nitrosamine
NNA	1-(*N*-Methyl-*N*-nitrosamino)-1-(3-pyridinyl)-4-butanal
NNK	4-(Methylnitrosamino)-1-(3-pyridyl)-1-butanone
NNN	*N*′-Nitrosonornicotine
LA-qPCR	Long amplicon-qPCR
HPRT	Hypoxanthine-guanine phosphoribosyltransferase
POLB	DNA polymerase β
HPB	4-Hydroxy-1-(3-pyridyl)-1butanone
FACS	Fluorescence-activated cell sorting
